# How We Apples Swim

**Published:** 1875-07

**Authors:** Hezekiah Jones

**Affiliations:** L. O., PLEB.


### HOW WE APPLES SWIM.

BY HEZEKIAH JONES, L. O., PLEB.

Ye Gods! and string beans, who would or could have conceived that there actually exists in our midst a scion of nobility. Yet undoubtedly such is the case. Lest some readers may be led astray, we would have you understand that we are not referring to some knight of the soil, who claims to be a sovereign over two by six feet of ground, who rises up in his might and power to deposit his token of sovereignity on election days, but a veritable descendent of either Shem, Ham or Japhet. (Just which deponent saith not, but we do vouch for one of them being his ancestor,) and that he, the living representative of either Shem, Ham or Japhet came into the world by right of inheritance a Bare-un. Thanks to the enterprising spirit that controls the destinies of that model periodical, The Penn. Journal of Dental Science, we have been permitted to peruse the biography of the aforesaid scion. Reader you will never know how we were completely overcome with mortification when we took up the above journal vol. II. No. 4, and there read the biography of our neighbor L. P. Meredith, M. D., D. D. S. We readjusted our specs looked again, and sure enough there it was L. P. Meredith, M. D., D. D. S., of Cincinnati, Ohio. We were ready to sink and gasped for a glass of water, a thousand recollections came trooping up, of how often we had passed by that sign never thinking that it represented anything more than the name of a respected neighbor dentist. And how time and again we had passed on the street and my chapeau was never lifted, how we had met, shook hands, spoke of the weather, business, etc., as ordinary equals. And now to be told in this modest way that the doctor descends "from an old and titled English family of the same name." "His father of the same name, when a child, came to America with his widowed mother, who was robbed on the voyage over of important papers and valuable portable property, which she was never able to regain, (the lachrymal fluid will flow as I read this sad part of the doctor's biography.)" We read further, that, "Her misfortunes were still further increased by the loss of her remaining means through the dishonesty of a business man, in whose hands she placed a considerable sum for investment." Oh dear! my handkerchief. What an amount of grace the doctor must be possessed of to be able to live through such trials. And just to think the poor disconsolate son's grandmother had nothing left but an annuity, which sufficed to keep her in just comfortable circumstances till her death, but then it is such a satisfaction to be told in the next sentence, that "A number of persons bearing the family name have occupied prominent political and professional positions."

The Bare-un's father, Philip Meredith, with a wisdom worthy of a Greely, concluded to go West, came to Ohio where he embarked in the jewelry business, but as that savored too much of the common plebian, he studied medicine, and strange to say "In 1835 he was married to Julia Sexton, a young lady of great beauties of person and mind." "She was the daughter of Col. Sexton, of Virginia, who held a distinguished position in the war of 1812." And who was also a man of mind as he too went West, locating a few miles north of Xenia.

It is so touching to be told that "About the year 1845, Philip Meredith finding that his health would not bear the exposure of a physican's life, turned his attention to dentistry, and removed to Cincinnati to engage in practice. Here he remained for twenty years, then retired to a recently purchased home at Yellow Springs, Ohio. Where he passed the remainder of his days."

The father of the son having failed in accumulating a superabundance of lucre for family use, the scion "felt it incumbent upon him to become his father's successor, although his tastes and previous studies inclined him to a pursuit altogether different."

We are utterly unable to comprehend the loss sustained by that vocation towards which genius inclined, but are consoled by the old adage, "As the twig is bent, the tree is inclined." And the world may even yet resound with praises sung in honor of the fame achieved by a direct descendant from an old and titled English family. "At an early age he exhibited more than ordinary ability for a youth, as a writer of both prose and poetry. And during the several years preceeding his beginning of the practice of dentistry, a good many of his productions were published." How marvellous, that one so gifted, so precocious, should live to man's estate, the only explanation given by our biographer, (and he knows) is that the baneful effects were counteracted by "his diffidence which was so great as to prompt him to offer his productions under a *uom de plume*, so that very few have ever known who was the author of some pieces that were very favorably noticed." Oh thou! most modest of men. Why were we prevented from rushing to thy shrine, that we might bow to the scintilations of thy genius. We implore thee to no longer hide they light under a bushel, but to let it shine forth and illumine the darkness by which we are surrounded on all sides. When we consider that "when but twelve years old, he wrote several chapters of a romance, but he was so mortified and vexed on going into the parlor one evening and receiving the congratulations of a company assembled, to whom his father was reading the discovered manuscript that he took the unfinished work from his hands and threw it into the fire." With feelings of reverence we behold how from very infancy, genius was embraced by modesty and still goes hand in hand through the scion's daily walk of life. Our heart beats with joy as we read further that the above "plot is still his favorite conception and that he intends to reproduce it some day." We timidly ask that it be not over a *nom de plume.*

The next evidence of a precocious intellect is his ability displayed in the game of chess, at sixteen years of age he won the championship and presidency of a club, conquering the most of famous players in the United States. Having met with but a single defeat he rests on his laurels.

Our biographer goes on to say (and he knows) that, "without any particular object in view, he arrived at that period of his life which has already been spoken of, when he felt it his duty to engage in the practice of dentistry. He has already acquired a fair knowledge of the profession without any special application." an illustration of the law of osmosis. However we are told that "In the year 1867 he graduated at the Philadelphia Dental College, and in 1871 he received his diploma from the Medical College of Ohio." There is, probably, no more marked an instance of considerable success in a pursuit altogether at variance with one's preferences.

We have not space to review the immense amount of work accomplished and detailed by the biographer. Our pulse is quickened as we read that "For several terms he lectured in the Cincinnati Medical College on Dental Medicine and Surgery. He now occupies the same position in the Ohio Medical College."

Now, oh reader, we did think the unprecedented prosperity of those institutions was due to the untiring zeal and labors of the distinguished gentlemen who constitute their faculties; but we are compelled to notice the fact that never until the Bare-un's connection with the Ohio Medical College has any institution west of the Allegheny mountains been able to graduate in one session so large a number of young men. From this time henceforth we shall never doubt that the unparallelled prosperiity of the Ohio Medical College is largely due to the fact of its having a lecturer on Dental Medicine and Surgery, who is a direct descendant of an old and titled English family.

However, we will not hestitate to make the assertion that none other than a direct descendant from an old and titled English family coupled with genius ever could have accomplished such marvellous results in so brief a space of time. His inventions have been numerous and of great value, but with characteristic modesty they have generally not gone far beyond his own office.

Our biographer goes on to say (and he knows) that, as "an operator no one has a more enviable local reputation. He takes great pride in novel and complicated operations, and he has frequent opportunities for performing them for persons of his own city, as well as for those that visit him from a distance; but he never has put himself in the way of extending his reputation in this respect." Thus we see at every turn the excessive modesty that prevents a fellow citizen from obtaining not only a national but world wide reputation.

Most devotedly do we hope that this descendant from an old and titled English family may overcome his exceedingly great diffidence and permit the world to enjoy the fruits of his labors, and in turn to offer homage to deserving genius, and finally when he shall be gathered to his fathers, erect a memorandum over his head that may be known and read by all men.*

*Those desiring copies of the above biography auto, apply to L. P. Meredith, M. D., D. D. S., etc., etc., Cincinnati, O.

